# ConStruct: Improved construction of RNA consensus structures

**DOI:** 10.1186/1471-2105-9-219

**Published:** 2008-04-28

**Authors:** Andreas Wilm, Kornelia Linnenbrink, Gerhard Steger

**Affiliations:** 1 Heinrich-Heine-Universität Düsseldorf, Institut für Physikalische Biologie, Universitätsstr. 1, D-40225 Düsseldorf, Germany

## Abstract

**Background:**

Aligning homologous non-coding RNAs (ncRNAs) correctly in terms of sequence and structure is an unresolved problem, due to both mathematical complexity and imperfect scoring functions. High quality alignments, however, are a prerequisite for most consensus structure prediction approaches, homology searches, and tools for phylogeny inference. Automatically created ncRNA alignments often need manual corrections, yet this manual refinement is tedious and error-prone.

**Results:**

We present an extended version of CONSTRUCT, a semi-automatic, graphical tool suitable for creating RNA alignments correct in terms of both consensus sequence and consensus structure. To this purpose CONSTRUCT combines sequence alignment, thermodynamic data and various measures of covariation.

One important feature is that the user is guided during the alignment correction step by a consensus dotplot, which displays all thermodynamically optimal base pairs and the corresponding covariation. Once the initial alignment is corrected, optimal and suboptimal secondary structures as well as tertiary interaction can be predicted. We demonstrate CONSTRUCT's ability to guide the user in correcting an initial alignment, and show an example for optimal secondary consensus structure prediction on very hard to align SECIS elements. Moreover we use CONSTRUCT to predict tertiary interactions from sequences of the internal ribosome entry site of CrP-like viruses. In addition we show that alignments specifically designed for benchmarking can be easily be optimized using CONSTRUCT, although they share very little sequence identity.

**Conclusion:**

CONSTRUCT's graphical interface allows for an easy alignment correction based on and guided by predicted and known structural constraints. It combines several algorithms for prediction of secondary consensus structure and even tertiary interactions. The CONSTRUCT package can be downloaded from the URL listed in the Availability and requirements section of this article.

## Background

Prediction of RNA structure as well as searches for homologues in large genomic sequence databases play a prominent role in the era of non-coding RNAs (ncRNAs). Structure prediction may provide insight into RNA function, and pattern-based database searches [1,2] may reveal new homologues, without the need for time-consuming experiments. Prerequisite for these predictions and searches as well as for inference of phylogeny [3-5] is the existence of an alignment of RNA homologues correct in terms of both sequence and structure. Sequence alignment tools like CLUSTALW [6] often fail to align ncRNA sequences correctly, especially when sequence homology drops below 60 % [7]. One reason is that ncRNA sequences evolve by compensatory base pair changes and ncRNA homologues are more conserved in structure than in sequence. For example, structural elements like thermodynamically extrastable tetraloops (UNCG, GNRA) share no sequence similarity and therefore cannot be correctly aligned by pure sequence alignment programs. Even structure alignment programs (e. g. DYNALIGN [8], FOLDALIGN [9], PMMULTI [10] or STEMLOC [11]) do not necessarily produce high-quality alignments under all conditions [7]. Moreover, these approaches are computationally extremely demanding, not only because they are based on simplified versions of the Sankoff algorithm [12]. Thus, automatically generated alignments often need to be corrected or refined by hand, which is a complex and tedious task. To ease this task a few sophisticated RNA alignment editors exist, e. g. 4SALE [13], SARSE [14] or S2S [15]. One of these tools is CONSTRUCT (**construct**ion of RNA **con**sensus **struct**ures; [16]), which is not only an RNA alignment editor but also allows for a variety of consensus structure predictions.

Here we present the completely revised and largely extended version of this tool and demonstrate some of its new features. CONSTRUCT allows for generation of RNA alignments, which are correct in terms of sequence and structure, by combining thermodynamic RNA structure prediction, several measures for covariation, and any alignment method. By applying this combination, typical shortcomings inherent to the single methods are eliminated; that is, the need of covariation for many, sufficiently divergent sequences is reduced, and the quality of thermodynamic predictions is enhanced. In contrast to tools, which predict a consensus structure automatically from a fixed alignment (e. g. RNAALIFOLD [17] or ILM [18]), CONSTRUCT allows for an interactive modification and optimization of the alignment. The user is able to modify the alignment similar to other RNA alignment editors [19,13,14]; the consequences of any alignment modifications are, however, immediately visible in a dotplot showing the probability of all base pairs of all RNA structures of the alignment; i. e., the user is guided during the alignment correction. In addition the user can account for sequence and structure constraints during the correction process. Afterwards optimal and suboptimal consensus structures and tertiary interaction can be predicted using a variety of built-in methods and displayed in several ways.

### ConStruct's approach to consensus structure prediction

**Figure 1:**
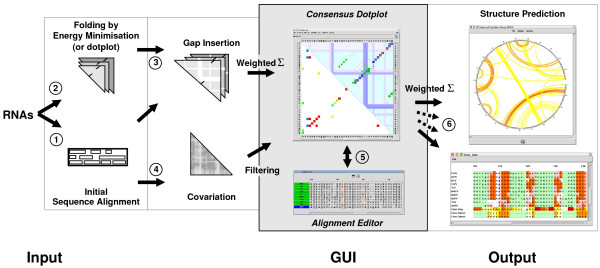
**Flowchart and graphical user interface of ConStruct**. Steps are numbered as in the text. The graphical user interface (grey part) shows results of a structural alignment for IRES regions of CrP-like viruses [45]; for a full view and further details of this alignment see Fig. 3. The main windows of CONSTRUCT are the "Consensus Dotplot" and the "Alignment Editor". The top-right triangle in the consensus dotplot shows thermodynamic base pairing probability of individual sequences (blue/green) and thermodynamic consensus matrix (red), the horizontal and vertical bars denote gaps; the lower-left triangle shows the MI (as a measure of covariance) normalized by pair entropy and a threshold of *t*_CV _= 50 % applied. Predicted structures may be displayed in several representations and formats. On the right side, two possible representations are shown. The Circles plot (upper window) shows the consensus structure as predicted by maximum weighted matching (MWM); consensus pairing probability is color-coded from white to red. The crossing arcs represent pseudoknots. Below the "Structural Alignment Output" is shown. From top to bottom: ten sequences [with background colors green for loops, red for consensus base pairs, pink for consensus base pair changes (covarying pairs), and white for non-base pairs in paired regions], the consensus sequence, and the consensus structure in bracket-dot notation and character-encoded (both with background colors from white to red proportional to sequence conservation resp. pairing probability). For an overview of colors used in CONSTRUCT see Table S4 in Additional file 1.

In the following we will describe the basic approach of CONSTRUCT (see Fig. 1).

**Figure 2:**
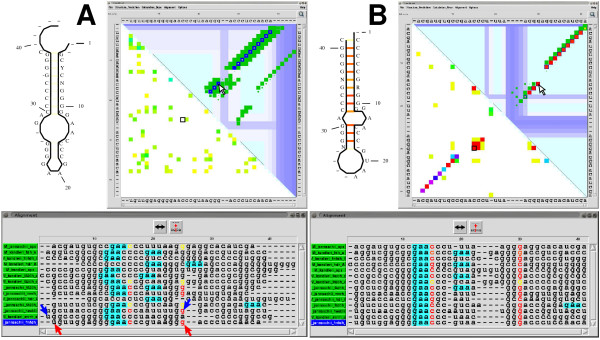
**Visualization of alignments by ConStruct**. An alignment of SECIS elements created by CLUSTALW (**A**) and after manual optimization/correction using CONSTRUCT (**B**). In both cases predicted consensus structures and CONSTRUCT's GUI are shown. For an overview of colors used in CONSTRUCT see Table S4 in Additional file 1. **Top left: **Corresponding drawings of consensus structures (annotated with the consensus sequence) generated by CONSTRUCT; consensus base pairing probability is color-coded from white to red. **Top right: **Corresponding dotplots: the base pairing probability of individual sequences (dark blue for the selected sequence M_janaschii_sps and green for others) is shown top-right in CONSTRUCT's main window; yellow to red dots show the consensus pairing probability; white to light blue bars denote gaps. The lower-left triangle shows the MI normalized by pair entropy with a threshold of *t*_CV _= 30 % in rainbow-colors from yellow to red. The cursors in A and B (arrow in thermodynamics part and black square in MI part) point to a similar position. **Bottom: **Corresponding alignment windows. Nucleotides participating in a base pair to which the cursor points in the dotplot are automatically highlighted [colored by pairing probability from *p *= 0 (black) to *p *= 1 (red)]. The motif **GAA **(turquoise background), which is conserved in the internal loop, has been highlighted using the built-in regular expression search. Clicking with a mouse button to position 3 and to position 25 of the last sequence (M_jannaschii_fmfdh_B; see red cursors) in the alignment editor selects this subsequence; clicking once with left or right mouse button to the double-headed arrow moves the subsequence towards 5' or 3' end, respectively, by one position; in the top-right dotplot the corresponding base pairs are automatically positioned. Similarly, clicking to a 5' and a 3' nucleotide of two different sequences (for an example see blue cursors) selects all corresponding subsequences from the sequence range; if none of the subsequences ends in a gap and all are followed by a gap, the subsequence range is moved towards the gap by clicking to the double-headed arrow.

1. First, an initial sequence alignment needs to be created by means of an alignment program of the user's choice (e. g. by a pure sequence alignment program like MAFFT [20], by a sequence+structure alignment program like STRAL [21] or STEMLOC [11], or by a pure structure alignment program like PROFILE-DYNALIGN [22]).

2. "Thermodynamic base pairing probability matrices" of all sequences in the alignment are automatically generated by means of a front-end program (CS_FOLD) to RNAfOLD [23]. An alternative to these thermodynamic approaches is creation of dotplots [24] with a minimum length of helices and thermodynamic weighting of their base-pair composition [25].

3. Gaps from the initial alignment (step 1) are inserted into the matrices (step 2), resulting in identically sized matrices that are superimposed, thus building a consensus matrix. Ideally, homologous base pairs should now possess identical positions. The base pair matrices for each single sequence as well as the consensus matrix are displayed in a graphical user interface (GUI; see green and blue dots in upper triangle of the consensus dotplots shown in Fig. 1 and 2). The probability of a thermodynamic consensus base pair (see red dots in the consensus dotplots) is calculated such that noise from individual base pairs not representing the consensus is reduced and over-representation of sequence families is avoided (for details see [16]). For an overview of colors used in CONSTRUCT see Table S4 in Additional file 1.

4. The new version of CONSTRUCT now allows to compute either the mutual information content (MI; [26]) or the RNAALIFOLD covariation score [17] to measure the amount of covarying positions (joined nucleotide substitutions, compensatory base pair changes). The results are displayed in the GUI (lower triangle of consensus dotplots in Fig. 1 and Fig. 2), can be filtered and normalized (see below), and are later on used in conjunction with the thermodynamic base pair matrices as the basis for consensus structure prediction. The MI at two aligned nucleotide positions *i *and *j *is defined as:

$${\text{MI}}_{ij}=\sum_{\text{X},\text{Y}}f_{ij}(\text{XY})\log_{b}\frac{f_{ij}(\text{XY})}{f_{i}(\text{X})\cdot f_{j}(\text{Y})}$$

where *f*_*i*_(X) and *f*_*j*_(Y) are the frequencies of the nucleotide types X ∈ {A,U,G,C}and Y ∈ {A,U,G,C} at aligned positions *i *and *j*, and *f_ij_*(XY) is the joint frequency of finding X at *i *and Y at *j*. In addition, he user may apply a normalization method [27], which enhances separation of truly correlated positions from background correlations. That is done by dividing the MI by the joint entropy

$$h_{ij}=\sum_{\text{X},\text{Y}}f_{ij}(\text{XY})\log_{b}f_{ij}(\text{XY}),$$

the upper bound of the MI. For statistical analysis of the MI, maximum likelihood or unbiased probability estimation [28] in nits (*b *= e) [26] or bits (*b *= 2) [29] are available.

In comparison to the MI, the covariation score implemented in RNAALIFOLD measures compensations in Watson-Crick and wobble base-pairs [17] only, which is advantageous during search for helices. The meaningfulness of this score can be further improved by taking stacking into account (as shown in [30]), which is also a built-in option of CONSTRUCT. For a comprehensive description of the RNAALIFOLD covariation measure we refer to [17] and [30].

5. The alignment of the sequences is displayed in a separate window (see alignment editor in Fig. 1). Position of base pairs from the dot plots is coupled with the position of the corresponding nucleotides in the alignment; i. e., pointing with the mouse to a consensus base pair highlights the corresponding base pairs in the alignment with a color from white to red according to the individual base pairing probabilities (see also Fig. 2); pointing to a base-paired nucleotide in the alignment changes the color of the corresponding base pair in the dot plot. A selected region of a single sequence or multiple sequences, with a gap at either side, may be moved with the mouse towards the gap, and the dot plots are updated correspondingly. Helices not superimposed are easily detectable. Thus, the user is guided during the alignment correction. These functions of the GUI are extremely helpful while correcting positions of structural elements, which were misaligned during the initial sequence alignment (step 1).

6. Consensus structure prediction is now based upon the weighted and filtered summation of the thermodynamic consensus dotplot (step 2) and the covariation dotplot (step 4), whereas the previous version of CONSTRUCT used the unfiltered thermodynamic consensus dotplot alone.

The probability *p*_*c *_of a thermodynamic consensus base pair at positions *i *and *j *is given by

$$p_{c}(i,j)={\left(\frac{\sum_{s=1}^{N}w_{s}\cdot p_{s}{\left(i,j\right)}^{1/3}}{\sum_{s=1}^{N}w_{s}}\right)}^{3}$$

where *N *is the total number of sequences and 0 ≤ *w*_*s *_≤ 1 is the user-defined weight of sequence *s*. This weighting can be used to avoid over-representation of a closely related sequence family in comparison to other sequences. The exponentiation helps to reduce low pairing probabilities from individual sequences.

The linear combination of the thermodynamic and the covariation pairing probabilities

$$\begin{array}{ccc} P_{c}(i,j) & = & w_{\text{TD}}\cdot \{\begin{array}{ll} p_{c}(i,j) & \text{if }p_{c}(i,j)>t_{\text{TD}}\\ 0 & \text{otherwise} \end{array}\\ & + & w_{\text{CV}}\cdot \{\begin{array}{ll} {\text{CV}}_{i,j} & {\text{if CV}}_{i,j}>t_{\text{CV}}\\ 0 & \text{otherwise} \end{array} \end{array}$$

allows for thresholds *t *and a relative weighting (*w*_TD _+ *w*_CV _= 1) of thermodynamics and covariation. The thresholds serve to further reduce the statistical noise and to suppress false positive base pairs and can be adjusted by the user. According to our experience, use of only thermodynamics with *w*_TD _= 1.0 and *t*_TD _= 0.03 already results in sensitive and specific predictions of secondary structures. The MI will only give additional information when the alignment contains many and divergent sequences. For tertiary structure predictions or detection of non-canonical base pairs [31], however, the MI must be reasonably high, since thermodynamic data alone are not sufficient.

Prediction of secondary structure is performed by dynamic programming [32] maximizing the weighted combination of the thermodynamic and covariation pairing probability. The new version of CONSTRUCT also allows to predict suboptimal structures [33,34]. More importantly, routines for predicting tertiary interactions (pseudoknots, triple pairs) by maximum weighted matching (MWM) [35] are now built into CONSTRUCT. Examples for both prediction types are given in the Results section.

**Figure 3:**
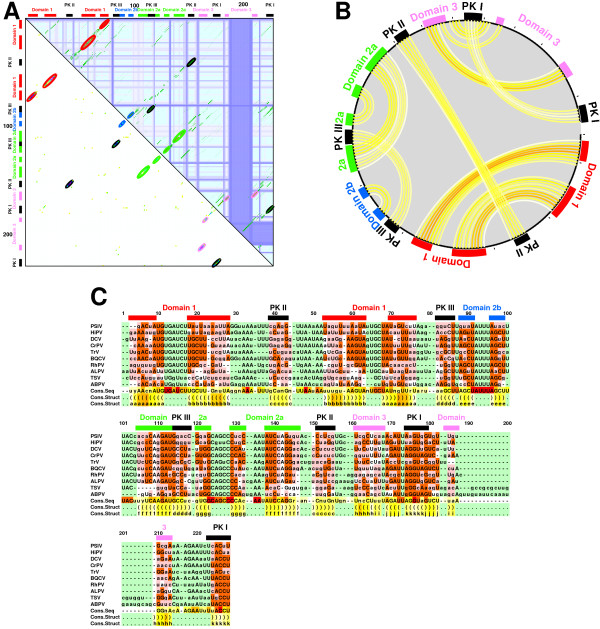
**Alignment of internal ribosomal entry sites (IRES) from CrP-like viruses [45]**. Subfigures A-C are created by means of CONSTRUCT using the RNAALIFOLD covariation score including stacking and parameters *w*_TD _= 0.5, *w*_CV _= 0.5, *t*_TD _= 0.03, and *t*_CV _= 0.15. Colored bars and labels are added in a graphics program according to the nomenclature given in [45]. The used color-coding is explained in Table S4 in Additional file 1. Sensitivity and specificity are above 90 % compared to the structures given in [47] and [46]; falsely predicted are only a few additional, non-contradictory base pairs, for example those labelled by "j" in figure part **C**. **A: **Dotplot; note that base pairs, which give rise to the pseudoknots (PK I-III), are present not only in the covariation plot (lower triangle) but also, with low probability, in the thermodynamics plot (upper triangle); i. e., they are part of suboptimal secondary structures. **B: **Circles plot. **C: **Structural alignment output.

The predicted consensus structures can be viewed directly or be stored in several formats (RNAVIZ [36], CONNECT [37] or RNAML [38]). Another newly added feature is the support for structure logos [39], which can be directly requested from within CONSTRUCT. Three different graphical representations are supported:

• The first representation is basically an alignment of the sequences, where the background of the nucleotides is colored according to the nucleotide's structural features (for examples see bottom of "Structure Prediction" in Figs. 1 and Fig. 3C). An additional text output describes the structural alignment in numerical form (numbers of base pairs, consensus base pair changes, mismatches, consensus base pairing probability, MI per helical position, and statistical significance of MI values by *χ*^2 ^test).

• The consensus structure–annotated by an individual sequence or the consensus sequence with or without alignment gaps–can be displayed in different, SQUIGGLES-like ways (for examples see structures in Fig. 2 left). Overlapping of helical regions may be avoided by user interaction; each helix is selectable by the mouse and may be rotated around the upstream loop. Base pair connections can be color annotated according to their probability.

• The consensus structure–annotated by an individual sequence or the consensus sequence–can be viewed as a circular graph (circles plot) with nucleotides as edges and base pairs connected by probability-annotated arcs (for examples see "Structure Prediction" in Fig. 1 and Fig. 3B). Such a plot allows for representation of tertiary interactions; i. e., crossing arcs denote pseudoknots. If chemical or enzymatic mapping data are available, the accessibility of nucleotides can be marked with small triangles.

The two time consuming steps 1 and 2 are executed only once, whereas the remaining steps are computed on the fly. Handling of less than 100 sequences of length below 500 nucleotides is done fluently on a standard desktop PC.

## Results and Discussion

The tool CONSTRUCT combines thermodynamic and statistical methods to predict the consensus structure of a set of homologous RNA molecules. In this respect it is similar to, for example, RNAALIFOLD [17] or ILM [18]. Yet, these programs use fixed alignments as input for structure prediction, whereas CONSTRUCT also allows to correct potentially incorrect alignments beforehand. CONSTRUCT's graphical user interface (GUI) guides the user in optimizing/correcting the alignment with respect to a consensus structure by displaying all base pair probabilities corresponding to the alignment in a consensus dotplot.

Most functions used in CONSTRUCT to extract a consensus structure from a given alignment are well known from the literature [32,33,26,35,17,30]. Given a reasonably good structural alignment CONSTRUCT is able to extract the correct consensus structure even without user intervention. Usually almost optimal results can be obtained by using thermodynamic pairing probabilities alone. If an alignment contains 5–10 sequences with an average pairwise sequence identity below ~70 %, then sensitivity as well as specificity [40] for secondary structure predictions are above 80 % (see Fig. S5 and Table S3 in Additional file 1; see also [30]). If additional information either from MI, normalized MI or RNAALIFOLD covariation is used, the mean accuracy of secondary structure predictions is not increased (see Additional file 1). A bonus is, however, the validation of predicted pairings by the statistical information. Nonetheless, covariation scores increase prediction quality for alignments with many sequences, especially when predicting unusual base pairs, and the MI is essential when predicting tertiary interactions (see Example 2 below).

### Summary of new features

Since CONSTRUCT's last release 9 years ago [16] the most striking new features are the display and use of several mutual information scores and the ability to predict tertiary interactions. The following types of measures for base pair covariation are now supported (see step 4 above): Mutual Information with optional pair-entropy normalization [26,27] or RNAALIFOLD with optional stacking [17,30], which are an essential requirement for the newly added prediction of tertiary interactions (see below). In previous versions, covariation was only used for a *χ*^2 ^test to verify predicted base pair positions. Now both types of covariation are displayed in the GUI. As covariation scores usually suffer from statistical noise and alignment errors, proper filtering and weighting is important when using it as a basis for structure prediction. CONSTRUCT now allows the user to adjust filter and weighting factors according to the displayed data and thus to make optimal use of it. Instead of using only thermodynamic data for structure prediction and validate this using statistics, we now combine the chosen covariance measure with the thermodynamic prediction and apply user-defined weights and thresholds. This filtered and weighted combination of both terms (see step 6 above) builds the basis for the following structure prediction step. On top of the standard Nussinov-style prediction of secondary structure prediction [32], we added the abilities to predict suboptimal consensus structures [33,34] using dynamic programming and tertiary interactions using the maximum weighted matching (MWM) procedures imatch and bmatch [35]; see also Figure 3. Some of these features are demonstrated in the following section.

On top of that, several new convenience features have been added, for example:

• input of alignments in most sequence formats *via *Eddy's SQUID package [41],

• color-coded regular expression searches (see e. g. the colored GAA-motif in the alignment window of Fig. 2),

• the built-in option to request RNA structure logos [39],

• removal of gaps from consensus structure drawings,

• a paned editor window to allow for simultaneous viewing and editing of 5' and 3' ends, and

• support for RNAplfold [42] to fold very long sequences locally.

CONSTRUCT version 3 is a reimplementation of the prior versions. Many time consuming Tcl functions–e. g. those responsible for update of the GUI (consensus dotplot) after alignment modification–have been ported to C and built into the custom Tcl interpreter to speed up the application. This GUI update step after alignment modification could take up to approximately 20 seconds for a big alignment of 450 sequences (moving 15 nucleotides at once); by using C-code (built into the interpreter) instead of interpreted Tcl-code the execution is speedup is roughly 15-fold.

### User optimization of a given alignment

#### Example 1: SECIS

"Selenocysteine insertion sequence" (SECIS) RNA elements from methanogenic organisms [43] form a stem-loop structure characterized by a relatively low degree of sequence conservation in the terminal helix (about 20 nt) and a higher degree of sequence conservation in the remaining part (about 14 nt). Accordingly, alignments created by standard sequence alignment programs are far from structurally correct. However, displaying such an alignment in CONSTRUCT (see dotplot in Fig. 2A for a ClustalW alignment) the correct consensus structure is readily identifiable: note the small yellow dots inside the blue squares and the "helix clustering" visible as a close accumulation of green diagonals in the upper triangle of the dotplot, which are not superimposed, i. e. not correctly aligned. Here, five of the 14 sequences are already superimposed in their structure (note the colored nucleotides in the alignment window). Furthermore, from the dotplot it is already obvious that most other, not-superimposed structures can be aligned with those by mainly horizontal adjustment of base-pair positions. A major shift is necessary only for the two hdr_A sequences (see the off-diagonal helices in the dotplot). The user is guided during this adjustment process–i. e. which of the sequences have to be selected, which nucleotides have to be moved in the alignment, etc.–by the direct interconnection between base pairs in the dotplot and corresponding nucleotides in the alignment editor. Additionally, the possibility to highlight certain nucleotides or motifs in the alignment window by means of regular expressions might be of help during the manual refinement stage. In case of the SECIS elements this is the conserved GAA in the internal loop (see for example the turquoise colored motif in the alignment windows in Fig. 2).

After the correction process (for a step-by-step example see Fig. S6 of Additional file 1) from the sequence alignment in Fig. 2A to the corrected alignment shown in Fig. 2B, the mean thermodynamic pairing probability of the terminal helix (see yellow to red dots in upper triangles of dotplots in Fig. 2) rises from 0.07 to 0.673, and the mean MI (see lower triangles of dotplots in Fig. 2) from 0.46 nits, which has a low significance according to *χ*^2 ^tests, to 0.86 nits, which is highly significant (all data computed inside CONSTRUCT). The alignment length is reduced by three nucleotides and all helices (green diagonals) except one are superimposed, thus building a consensus helix (red diagonal). The exceptional helix belongs to sequence M_kandleri_hdr_A, for which at least two different alignments are possible; details of these alternatives are shown in Additional file 1 (Fig. S2). A decision about such cases is left to the user and/or further (experimental) input.

#### Example 2: Tertiary interactions in CrP-like viruses

The second example shows prediction of a consensus structure including pseudoknots. In general the prediction of pseudoknots, triple base pairs, or any non-Watson-Crick pairs is difficult in comparison to prediction of a secondary consensus structure because thermodynamic predictions are usually limited to pseudoknot-free structures. Tertiary helices are, however, quite often part of suboptimal secondary structures included in the partition function prediction. Furthermore, base pairs in tertiary structural elements are quite often more conserved in sequence than the isosteric Watson-Crick and wobble base pairs [31] in secondary structural elements. This higher degree of sequence conservation leads to a better (sequence) alignment in corresponding regions. Anyway, pairings predicted by the partition function and/or helix dotplots (step 2) in combination with MI or RNAALIFOLD's covariation measure (step 4) followed by MWM prediction (step 6) results–similar to secondary structure prediction–in prediction with about 80 % sensitivity and specificity (for a detailed analysis see [25]).

Cricket paralysis-like viruses use an internal ribosomal entry site (IRES) for an 5'-end-independent pathway of translation initiation [44-48]. This IRES (with lengths up to 200 nt) contains three pseudo-knots, which are noticeable as crossing lines in the circle plot (Fig. 3B). The alignment contains 10 sequences with an average pairwise sequence identity of about 50 % and is slightly modified by means of CONSTRUCT from that given in [49]. The pseudoknot helices (encircled by black lines in Fig. 3A, top right triangle) are already visible in the aligned "thermodynamic base pairing probability matrices", but are much more prominent in covariation plots (Fig. 3A, left bottom triangle). Summation of pairing probabilities from thermodynamics predicted matrices and covariation plots followed by MWM structure prediction leads to a consensus structure depicted as circular graph in Fig. 3B and as structure-annotated alignment in Fig. 3C. Most predicted pairings are in accordance with those given in the literature [46,47]; sensitivity and specificity are 93 and 90 %, respectively, compared to the structure given in [47] and 92 % compared to the structure given in [46] (computed with compare.pl [25]). CONSTRUCT predicts only additional, non-contradictory base pairs; examples are two additional pairs in the proximal helix of Domain 1 and three to four additional pairs in the hairpin of Domain 3.

### Application to reference alignments of BRAliBase

The first comprehensive RNA alignment benchmark (BRALIBASE II; [7]) used reference alignments created from four alignments taken from the RNA family database Rfam [50,51]. The reference alignments were compiled in such a way that each contained five sequences, which were equally distributed across the available range of sequence identity. For each of these four RNA families we took the reference alignment which is hardest to align–i. e., it has the lowest sequence identity–and optimized it using CONSTRUCT. Reference independent quality measures of the original BRALIBASE alignment and the alignment corrected with CONSTRUCT are listed in Table 1. While the sequence identities of the original and optimized/corrected alignment remain almost the same, the structural conservation is increased clearly during the optimization. The structurally misaligned regions are easily identified in CONSTRUCT's consensus dotplot display (see Table S1) and can easily be corrected in a few steps (see also SECIS example in previous section and Fig. 2).

**Table 1:** Optimizing reference alignments of BRAliBase.

	BRALIBASE			CONSTRUCT		
		
Alignment	APSI	SCI		APSI	SCI	
			
		cov	w/o cov		cov	w/o cov
g2intron/aln51	0.46	0.55	0.42	0.45	0.75	0.58
rRNA/aln74	0.49	0.68	0.53	0.50	0.81	0.61
tRNA/aln27	0.35	1.19	0.73	0.36	1.22	0.76
U5-RNA/aln41	0.50	0.61	0.42	0.50	0.65	0.49

For each RNA family used in BRALIBASE [7] we extracted the hardest alignment, i. e. the alignment with lowest sequence identity (measured as average pairwise sequence identity; APSI), and corrected it using CONSTRUCT. Quality measures for the original BRALIBASE alignments and the alignment optimized with CONSTRUCT are shown: sequence identity is measured as APSI, structural conservation as structure conservation index (SCI) with (cov) and without (w/o cov) covariation term, respectively. By using CONSTRUCT, the structural conservation of the alignments is clearly enhanced while maintaining sequence conservation. Dotplots of the alignments are shown in Table S1 of Additional file 1.

As the BRALIBASE alignments were re-compiled automatically from bigger Rfam alignments they naturally contain some errors. We wish to note however that the BRALIBASE alignments are generally of good quality. A recent publication of another sophisticated editor, SARSE [14], showed that more than 10 % of all entries in the Rfam database are either misaligned (for an example see Fig. S8) or their structure is inconsistently annotated. Thus one should be aware of the limitations when using those alignments as a reference for alignment benchmarking. A benchmark consisting of hand curated alignments supported by predicted and/or known structures has yet to be compiled. RNA alignment editors like SARSE, S2S and CONSTRUCT would be crucial for this task.

## Conclusion

RNA alignment and consensus structure prediction is still a circular problem: A consensus structure needs to be known to create a high-quality alignment, but a high-quality alignment is prerequisite for consensus structure prediction. Thus, despite recent advances on this field [52-55] the need for RNA alignment editors which allow manual refinement based on structural properties is still there. These editors are still widely used and become increasingly sophisticated (see e. g. [13] and [14]). Automatically created RNA alignments and corresponding consensus structure prediction can be optimized in most cases (see this manuscript and [14]).

Our tool, CONSTRUCT, guides the user in correcting structurally misaligned regions. Once the initial alignment is refined, CONSTRUCT is able to predict secondary as well as tertiary consensus structures with high sensitivity and specificity. CONSTRUCT has already been described as an effective and "most elegant" [56] tool for structure alignment generation and RNA structure prediction. One of its strength is the "elaborate GUI" [56] that allows for easy identification and correction of structurally misaligned regions, guides the user in correcting an initial RNA sequence alignment, and allows for setting proper weight and threshold parameters for consensus structure prediction. Structurally misaligned regions are readily identifiable in the thermodynamic consensus dotplot and can be corrected by means of the built-in alignment editor. The example shown in Fig. 2 is typical in the sense that with a pure sequence alignment the "correct" consensus structure is already detectable in the CONSTRUCT dotplot and necessary corrections of the initial alignment are quite obvious.

The gold standard approach for RNA consensus-structure prediction–Comparative Sequence Analysis using covariation and MI [28,57]–needs many sequences that have to be nearly perfectly aligned, which in turn is almost impossible for most sequence sets. Even given the perfect and large alignment, predictions only based on MI often suffer from non-informative columns (due to either too high sequence conservation or too many gaps) in the alignment. Purely thermodynamic based prediction methods are usually fairly reliable and allow for structure prediction from a few (in the extreme case one) sequences, and gain specificity and sensitivity when more sequences are added. Yet (standard) thermodynamic approaches alone cannot detect tertiary interaction or non-canonical base pairs. By using the (pair-entropy normalized) MI, which makes explicitly no use of base pairing rules, or the RNAALIFOLD covariation function (including stacking), which acknowledges consistent base pair mutations, CONSTRUCT is also able to predict non-canonical base pairs and tertiary interactions (for example see Fig. 3C). The prediction of tertiary interactions or at least certain types of pseudoknots could in principle be enhanced by including data into CONSTRUCT from structure-prediction programs other than RNAfold (for a review of alternatives see [58]).

From the results presented here–and our experience over the last years using CONSTRUCT–we propose the following approach for building up an RNA alignment for consensus structure prediction by means of CONSTRUCT:

1. Usually few sequences are initially available.

2. By pure sequence search (like BLAST) one could try to find more homologues of the sequence(s) from step 1. (Due to the sequence search the found homologues will be closely related to the already known sequences. For an overview and benchmark of selected RNA search tools see [59].)

3. Create an alignment of the sequences using an alignment program of your choice and depending on length and number of sequence; for example MAFFT [20], STRAL [21] or STEMLOC [11]; see [7,60] for benchmarks. This preliminary consensus structure should be checked for consistency by means of CONSTRUCT using only thermodynamics.

4. With help of the preliminary consensus structure, creation of either a pattern or a covariance model (CM) is possible. Both allow to search–for patterns with programs like PatScan [61] or HyPa [1] and for CMs with programs like infernal [62] or RSEARCH [2]–more specifically for further members of the RNA group under inspection.

Alternatively, reiterate from step 2.

5. Check the refined model for consistency with CONSTRUCT using thermodynamics and covariation analysis. If this gives new information–especially in terms of tertiary interactions and/or base triples–reiterate from step 4, otherwise this final model could be refined further by verification from wet lab experiments.

In case of additional structural knowledge, for example from chemical or enzymatic mapping [63,64], the initial structure prediction by RNAFOLD can accordingly be constrained (see RNAfold manual and [65]) and thus incorporated into CONSTRUCT. If even information on the three-dimensional structure of one of the sequences from the set is available from X-ray or NMR analysis, the use of S2S [15] in addition to CONSTRUCT is advantageous.

## Methods

### Sensitivity and Specificity

Given a reference and a consensus secondary structure predicted by CONSTRUCT, we use the script compare_ct.pl [40] to compute sensitivity ("hit rate") and specificity (selectivity). In case of tertiary structures we use the corresponding script compare.pl [25].

### Alignment Scores

For computation of the structure conservation index (SCI) we used scif [60] and for the computation of the average pairwise identity we used alistat from Sean Eddy's SQUID package [41].

## Availability and requirements

CONSTRUCT version 3 is based on a previously published version [16] and has been rewritten and largely extended. The underlying interpreter (Tcl/Tk 8.4) was extended to speed up the application; the installation process uses the GNU autotools. We successfully tested CONSTRUCT under several Linux distributions (Ubuntu, Debian, SuSE, Red Hat Fedora) as well as under Mac OS X (using Fink). Input of sequences is format independent due to use of the SEQIO package [66]. Graphics output is produced in PostScript. Various output formats for structures and further processing are supported (e. g. RNAML, connect etc.). The CONSTRUCT package (source and Debian package) including a manual can be downloaded at .

## Competing interests

The authors declare that they have no competing interests.

## Authors' contributions

All authors contributed to the described tool and approved the final manuscript.

## Supplementary Material

Additional file 1 For supplementary material see accompanying PDF file, which is also available at Click here for file
