# Supplementary material for: ConStruct: Improved construction of RNA consensus structures
Source: BMC Bioinformatics. 2008 Apr 28;9:219. doi: 10.1186/1471-2105-9-219 (PMC2408607; doi:10.1186/1471-2105-9-219)
Supplement: Additional file 1 — For supplementary material see accompanying PDF file, which is also available at [file 1471-2105-9-219-S1.pdf]

# Additional file 1

**to**

# ConStruct: improved construction of RNA consensus structures

Andreas Wilm, Kornelia Linnenbrink, and Gerhard Steger

Heinrich-Heine-Universität Düsseldorf, Institut für Physikalische Biologie, Universitätsstr. 1, D-40225 Düsseldorf, Germany

Email:
A. W. - wilm@biophys.uni-duesseldorf.de;
K. L. - linnenbr@biophys.uni-duesseldorf.de;
G. S.* - steger@biophys.uni-duesseldorf.de;

*Corresponding author

## Table S1 - Optimizing reference alignments of BRAliBase.

Below are depicted dotplots of alignments as contained in BRALiBASE (left column) and after manual optimization using CONSTRUCT (right column). Corresponding alignments are shown in Fig. S1; for further details see Table 1.

BRALiBASE CONSTRUCT

**g2intron / aln51**

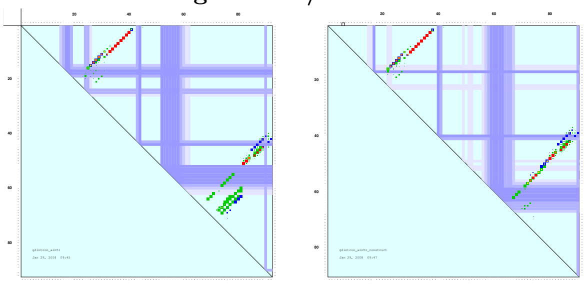

**rRNA / aln74**

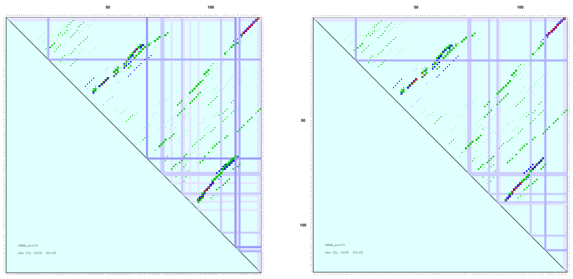

**tRNA / aln27**

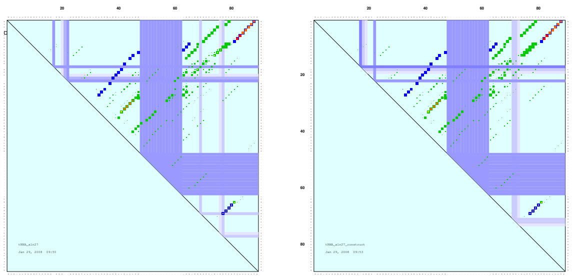

**U5 / aln41**

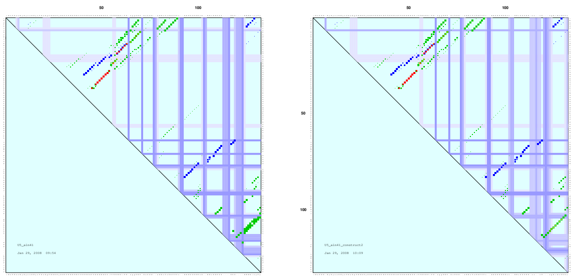

## g2intron / aln51

```
>X03834.1-1418_1494
gggagccggaugaga---agaaac-ucucauguccgguucuguaguagaga---------
--uggaauagggaaauaaccaucaacuauaac
>U77945.1-1848_1919
aaccgccguguacg-----gaac---cguacgcacggugug--ugagagg---------
acggcgggugugaauccgccuccuacucg-au
>L36897.1-2659_2743
gaaagccguaugaugg---gaaa-cuaucacguacgguuugg--gaaaggcucuuuaaca
cguggcaacauagguuaauuugcuauuuc-au
>X04465.1-4746_4826
uuaagccauacaga--guugaaaa-uaucauauaugguuuuca-agggggg-------aa
aaagaauuauaaaaucuaaaaccuauccuaau
>Y18999.2-2881_2957
uugagccggaugaggg---gcga-cucucauguccgguucuu--aggggac--------g
gcggcgcaguaaugcgcugcuguuacccg-ac
```

```
>X03834.1-1418_1494
gggagccggaugagaa-gaaacucucauguccgguucuguaguagagaug
gaaua------------gggaaauaaccaucaacuauaac
>U77945.1-1848_1919
aaccgccguguacg---gaac--cguacgcacggugug--ugagaggac
ggcgggu----------gugaauccgccuccuacucgau-
>L36897.1-2659_2743
gaaagccguaugaugg-gaaacuaucacguacgguuugg--gaaaggcuc
u-uuaacacguggcaacauagguuaauuugcuauuucau-
>X04465.1-4746_4826
uuaagccauacagaguugaaaauaucauauaugguuuuc-aaggggggg-a
aaaaga-------auuauaaaaucuaaaaccuauccuaau
>Y18999.2-2881_2957
uugagccggaugaggg-gcgacucucauguccgguucuu--aggggacgg
-cggcgca--------guaaugcgcugcuguuacccgac-
```

## rRNA / aln74

```
>X01000.1-1_121
acguacggccauauccgagacacgcguaccggaacccauuccgaauuccgaagucaagcg
uccgcgag-uuggguuaguagucuggugagggaucacaggcgaaccccaa-u-gcugua
cguc
>L08503.1-3_117
cuuggugguuauggcggaac-gccugcacccgaucccaucccgaacucggccgugaaacg
uuccagcg-cugaug--guacuuug-ucu-uaa-ggcacgggag-aguaag-ucgcugcc
aggu
>M21086.1-8_123
--acccggccauagcggccg-ggcaacacccggacucaugucgaacccggaaguuaagcc
ggccgcguuggggggaugcug-uggggucccgcgaggccccgcagcgcccccaa--gccggg
au--
>X05041.1-3_118
cuuggcgaccauaguguugu-ggacccaccugacucuaugccgaacucagaagugaaacg
uaauagcg-ccgaug--guagugug--gggcuuccccaugugag-aguagg-acaucgcc
aggc
>X05528.1-1_121
acauucggccauacuugguagauaugcgccuuaucccaucccgaacugagaagcuaaguc
uccauagg-cccagggaguacugcggucagggaugacgcuggaaaucuggg-u-gcugaa
uguu
```

```
>X01000.1-1_121
acguacggccauauccgagacacgcguaccggaacccauuccgaauuccg
aagucaagcguccgcgaguuggguuaguagucuggugagggaucacaggc
gaaccccaau-gcuguacguc
>L08503.1-3_117
cuuggugguuauggcggaac-gccugcacccgaucccaucccgaacucgg
ccgugaaacguuccagcgcugaug--guacuuug-ucu--uaaggcacgg
gaga-guaagucgcugccaggu
>M21086.1-8_123
--acccggccauagcggccg-ggcaacacccggacucaugucgaacccgg
aaguuaagccggccgcguuggggggaugcuguggggucccgcgaggccccgc
agcgcccccaa-gccgggau--
>X05041.1-3_118
cuuggcgaccauaguguugu-ggacccaccugacucuaugccgaacucag
aagugaaacguaauagcgccgaug--guagugug-ggg-cuuccccaugu
gaga-guaggacaucgccaggc
>X05528.1-1_121
acauucggccauacuugguagauaugcgccuuaucccaucccgaacugag
aagcuaagucuccauaggcccagggaguacugcggucagggaugacgcug
gaaaucugggu-gcugaauguu
```

## tRNA / aln27

```
>AL391016.1-2459_2530
gcucacaugguccagu-gguu-aagacucaucguuguggccgaugcg-------------
--acccagguucgauuccugguguggca
>J01390.1-12505_12576
uauguugucgacuaaucggu--aagucauaaauuuuugguauuuaua-------------
--uuggguguucgagucgccccaacauaa
>M86496.1-1_68
guuaauguagcuuaaa-auu--aaagcaaggcacugaaaaugccuag-------------
--augagu-auauua--acuccauaaaca
>AF347001.1-16015_15948
cagagaauaguuuaaa-uu---agaaucuuagcuuuggguggcuaaug-------------
--guggag-uuaaaga-cuuuuucucuga
>X61065.1-77_164
ggagcgauacucaaga-ggccgaagagggcgcacugcuaacgcguuagacgguuaacccc
gugcgaggguucaaaucccucucgcuccg
```

```
>AL391016.1-2459_2530
gcucacaugguccagu-gguu-aagacucaucguuguggccgaugcg---
------------acccagguucgauuccugguguggca
>J01390.1-12505_12576
uauguugucgacuaau-cggu-aagucauaaauuuuugguauuuaua---
------------uuggguguucgagucgccccaacauaa
>M86496.1-1_68
guuaauguagcuuaaa--auu-aaagcaaggcacugaaaaugccuag---
------------augaguau---auuaacuccauaaaca
>AF347001.1-16015_15948
cagagaauaguuuaaa---uu-agaaucuuagcuuuggguggcuaaug---
------------guggaguu--aaagacuuuuucucuga
>X61065.1-77_164
ggagcgauacucaaga-ggccgaagagggcgcacugcuaacgcguuagac
gguuaaccccgugcgaggguucaaaucccucucgcuccg
```

## U5 / aln41

```
>AL627223.23-85084_85197
cugcuugguuugcucuucaggucuuguaaaucuuucaccuuuuauaaaagauuuc--ugg
agaaggaagc-acucuggaagucuuaacu---gauuuuugu---ggcugcuu----cuu-
----ggugguuc
>AB017066.1-16062_15941
agccgu-gcagugagggcauagcgaacuauucuuucgccuuuuacuaaagaauaucgugu
ucu-cucugcuuuuaa--acggcauacgcc--uauuuuuggaaagguuuauu----acua
aauaauaaaccu
>AE014823.1-125450_125571
ggugugugguacuuacuacauaacgaaucaaucuuucgccuuuuacuaaagauugccgugu
agu-aaguau-guuaa-auacaauauaccacgaauuuuuguc--gcgccuauuaaguu--
---aggugcuca
>AP006390.1-45876_45755
agccuc-gcaugaagcacaaagcgaacuauucuuucgccuuuuacuaaagaauaccgugu
gcu-uguugc-aacaa--gaggcauacgcc--auuuuuugaa-gggauucuucca-uuug
ga-aggaacccu
>AC004395.1-1698_1591
gugug-ugggccuagcuca----gaauccauuucuugccuuuuaccagagauggccgugg
gcc-acgcaa-aauac---ccuacuaccu---aaucuuugga--aucaucua----uc--
---gacauaguc
```

```
>AL627223.23-85084_85197
-----cugcuugguuugcucuucaggucuuguaaaucuuucaccuuuuau
aaaagauuu--cuggagaaggaagc-acucuggaagucuuaacug---au
uuuugu---ggcugcuu----cuu-----ggugguuc
>AB017066.1-16062_15941
-----agccgu-gcagugagggcauagcgaacuauucuuucgccuuuuac
uaaagaauaucguguucu-cucugcuuuuaa--acggcauacgcc--uau
uuuuggaaagguuuauua---cuaaa-uaauaaaccu
>AE014823.1-125450_125571
-----ggugugugguacuuacuacauaacgaaucaaucuuucgccuuuuac
uaaagauugccguguagu-aaguau-guuaa-auacaauauaccacgaau
uuuuguc--gcgccuauuaaguu-----aggugcuca
>AP006390.1-45876_45755
-----agccuc-gcaugaagcacaaagcgaacuauucuuucgccuuuuac
uaaagaauaccgugugcu-uguugc-aacaa--gaggcauacgcc--auu
uuuugaa-gggauucuuccauuugga--aggaacccu
>AC004395.1-1698_1591
-----gugugu-gggccuagcuca----gaauccauuucuugccuuuuac
cagagauggccgugggcc-acgcaa-aauac---ccuacuaccua---au
cuuugga--aucaucua----uc-----gacauaguc
```

**Figure S1 - Sequence files from BRAliBase used in Table S1 and Table 1.**
A text file containing these alignments is available at http://www.biophys.uni-duesseldorf.de/construct3/paper2007suppl/Alignments_Tab1.txt .

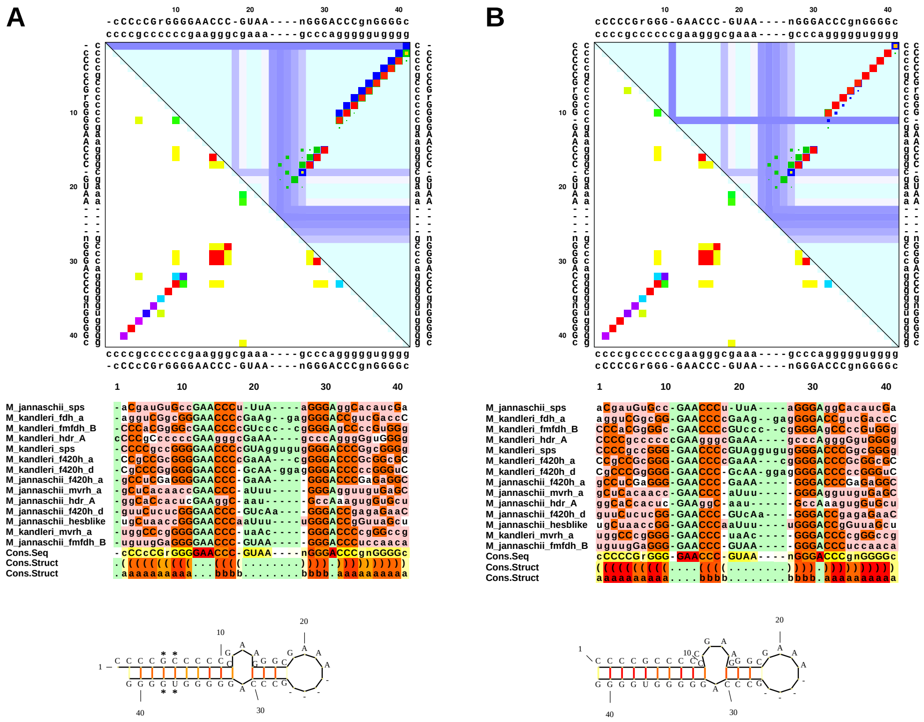


**Figure S2 - Alternative SECIS alignments.**

**Top:** ConStruct consensus dotplots. Base pairing probability of individual sequences (dark blue for the selected sequence M_kandleri_hdr_A and green for others) and consensus pairing probability (yellow to red dots) are shown in the upper right triangle. The MI normalized by pair entropy with a threshold of $t_{\mathrm{CV}} = 30\,\%$ is shown in the lower triangle. The dotplots are labelled with the sequence of M_kandleri_hdr_A and with the consensus sequence.

**Center:** Structural alignment output from ConStruct. Shown are the sequences [with background colors green for loops, red for consensus base pairs, pink for consensus base pair changes (covarying pairs), and white for non-base pairs in paired regions], the consensus sequence (with background colors from white to red proportional to sequence conservation), and the consensus structure in bracket-dot notation and character-encoded (both with background colors from white to red proportional to pairing probability).

**Bottom:** Schematic drawings of consensus structure generated by ConStruct (annotated with the sequence of M_kandleri_hdr_A). Consensus base pairing probability is color-coded from white to red. Positions that are paired in all sequences except M_kandleri_hdr_A are marked by asterisks.

**A:** An alignment that results for sequence M_kandleri_hdr_A in a 8 bp terminal helix and an internal loop, which coincides in size and sequence with all other sequences.

**B:** An alignment that results for sequence M_kandleri_hdr_A in a 10 bp terminal helix but an unusual insertion of a C in the internal loop.

## A

```
> M_jannaschii_sps
-acgaugugccgaacccu-uua----agggaggcacaucga

> M_kandleri_fdh_a
-aggucggcgggaaccccgaag--gaggggaccgucgaccc

> M_kandleri_fmfdh_B
-cccacggggcgaaccccguccc--cggggagccccguggg

> M_kandleri_hdr_A
ccccgccccccgaagggcgaaa----gcccagggggugggg

> M_kandleri_sps
-ccccgccggggaaccccguaggugugggggacccggcgggg

> M_kandleri_f420h_a
-ccgccgcggggaaccccgaaa---cggggacccgcggcgc

> M_kandleri_f420h_d
-cgcccgggggaaccc-gcaa-ggaggggacccccggguc

> M_jannaschii_f420h_a
-gccucgaggggaaccc-gaaa-----gggacccgagaggc

> M_jannaschii_mvrh_a
-gcucacaaccgaaccc-auuu-----gggagguugugagc

> M_jannaschii_hdr_A
-ggcaccacucgaaggc-aau------gccaaaguggugcu

> M_jannaschii_f420h_d
-guucucucgggaaccc-gucaa----gggaccgagagaac

> M_jannaschii_hesblike
-ugcuaaccgggaacccaauuu----ugggaccgguuagcu

> M_kandleri_mvrh_a
-uggcccgggggaaccc-uaac-----gggaccccgggccg

> M_jannaschii_fmfdh_B
-uguuggaggggaaccc-guaa-----gggacccuccaaca
```

## B

```
> M_jannaschii_sps
acgaugugcc-gaacccu-uua----agggaggcacaucga

> M_kandleri_fdh_a
aggucggcgg-gaaccccgaag--gaggggaccgucgaccc

> M_kandleri_fmfdh_B
cccacggggc-gaaccccguccc--cggggagccccguggg

> M_kandleri_hdr_A
ccccgccccccgaagggcgaaa----gcccagggggugggg

> M_kandleri_sps
ccccgccggg-gaaccccguaggugugggggacccggcgggg

> M_kandleri_f420h_a
ccgccgcggg-gaaccccgaaa---cggggacccgcggcgc

> M_kandleri_f420h_d
cgcccggggg-gaaccc-gcaa-ggaggggacccccggguc

> M_jannaschii_f420h_a
gccucgaggg-gaaccc-gaaa-----gggacccgagaggc

> M_jannaschii_mvrh_a
gcucacaacc-gaaccc-auuu-----gggagguugugagc

> M_jannaschii_hdr_A
ggcaccacuc-gaaggc-aau------gccaaaguggugcu

> M_jannaschii_f420h_d
guucucucgg-gaaccc-gucaa----gggaccgagagaac

> M_jannaschii_hesblike
ugcuaaccgg-gaacccaauuu----ugggaccgguuagcu

> M_kandleri_mvrh_a
uggcccgggg-gaaccc-uaac-----gggaccccgggccg

> M_jannaschii_fmfdh_B
uguuggaggg-gaaccc-guaa-----gggacccuccaaca
```

**Figure S3 - Sequence files used in the alternative SECIS alignments of Fig. S2.**

Corresponfing sequence files are available at http://www.biophys.uni-duesseldorf.de/construct3/paper2007suppl/secis_methanococcus_construct_A.vie and http://www.biophys.uni-duesseldorf.de/construct3/paper2007suppl/secis_methanococcus_construct_B.vie , respectively.

```
>PSIV
----gacuaugugaucuuauuaaaauuagguuaaauuucgagg--uuaaa
aauaguuuuaauauugcuauagucuuaga--ggucuuguauauuuauacu
uaccacacaagauggacc-ggagcagcccucc--aauaucuaguguac--
ccucgugc--ucgcucaaacauuaaguggugu--ugu-------------
---------gcgaaa-agaaucucacuu
>HiPV
--gaaaaugugugaucugauuagaaguaagaaaa-uu-ccuag--uua-u
aauauuuuuaauacugcuacauuuuuaag--acccuuaguuauuuagcuu
uaccgcccaggaugggu-gcagcguuccugc--aauauccagggcac--
cuaggugc--agccuuguaguuuuaguggacu-uua--------------
---------ggcuaa-agaauuucacua
>DCV
-guuaag-augugaucuugcuu-ccuuauacaau-uuugagagg-uuaau
aagaagg-aaguagugcuau-cuuaauaau-uagguuaacuauuuaguuu
uacuguucaggaugccua-uuggcagccccau--aauauccaggacac-c
cucucugc--uucuuauaugauuagguuguca-uuu--------------
---------agaaua-agaaaauaaccu
>CrPV
---caaaaaugugaucuugcuu-guaaauacaau-uuugagagg-uuaau
aaauuac-aaguagugcuauuuuuguauu--uagguuagcuauuuagcuu
uacguuccaggaugccua-guggcagccccac--aauauccaggaagc-c
cucucugc--gguuuuucagauuagguaguc--gaaa-------------
---------aaccua-agaaauuuaccu
>TrV
--uugacuaugugaucuugcuu-ucguaauaaaa----ucuguacauaa-
aagucga-aaguauugcuauaguuaagguu-gcgcuugccuauuuaggca
uacuucucaggauggcgc-guugcaguccaac--aagauccagggacugu
acagaau---uuuccuauaccucgagucggguuu----------------
---------ggaauc-uaagguugacuc
>BQCV
--ccaacaaugugaucuugcuu-gcggaggcaaaauuugcacaguauaa-
aaucugc-aaguagugcuauuguuggaau--caccguaccuauuuagguu
uacgcuccaagaucgguggauagcagcccuauc-aauaucuaggaga-ac
ugugcua---uguuuagaagauuagguagucu--cua-------------
---------aacaga-acaa-uuuaccu
>RhPV
--aguguugugugaucuugcgc-gau-----aaa-ugcugacg--ugaa-
aa-cguu-gcguauugcuacaacacu------ugguuagcuauuuagcuu
uacuaaucaagacgccg-ucgugcagcccaca--aaagucuagau----a
cgucac--aggagagcauacgcuaggucgcgu--ugac------------
---------uauccu-uauauaugaccu
>ALPV
aauuacuaauuugaucuuu--agguua---uaauguuaggacua--uaaa
aauuagcuu--aa-ugcauuuaguaauuua-aggcuuaguuauuuaacuu
uacuuaucaagauggc-cguuggcagcccc-acgaa-aucuagau----u
aguccgaauguccuauuuugauuagguggguca--gau-------------
---------agguca--gaaacucaccu
>TSV
-cuccauguauugguuacccaucugcaucgaaaac---ucuccgaacacu
aggugcaguaag---gcuuucauggag-----ugguuugcuauuuagcg-
uac------guguacca--uaggcagccccaa--aa-acac-guguga--
ggaga--aagucccagucacuuugggcaaaguagaca---gccgcgcuug
cguggu---gggacuu-aauuaaugccu
>ABPV
----cacaacaugguuacccauag-auugaggaaauuucc-aa--uaaac
ucaguauuaagg---cuuguuguguuggac-aaggugcccuauuuagg--
-----gug-aggagccuuacuggcagccccagugaa-uccu-cca-----
uuggaua--ggaacagcuauauugguaguuguagcaguuguauucaaau
gaaugcagcguuccgaaauaucauaccu
```

**Figure S4 - Sequences of internal ribosomal entry sites (IRES) from CrP-like viruses used to produce Fig. 3.** A corresponding file is available at http://www.biophys.uni-duesseldorf.de/construct3/paper2007suppl/crp-like-virus-ires.vie .

## 1 Structure prediction from fixed alignments

To test ConStruct's ability to extract a consensus structure from a given alignment, we selected "seed" alignments from the Rfam database v. 7.0 [1, 2] as references according to the following criteria:

- The alignment has a sequence length below 500 nt; with longer sequence lengths the quality of thermodynamically predicted structures drops (see e. g. [3]).
- The alignment contains less than 40 sequences; larger homologous ncRNA sequence sets are rarely available to a user.
- The sequence homology (measured as average pairwise sequence identity; APSI) is below 70 %; this limit should enhance the likelihood for compensatory base pair changes.
- The Rfam structure annotation does not contain tertiary interactions, which can not be handled by the script `compare_ct` [4], which we use to determine the structure prediction quality.

We applied ConStruct (without any user intervention) and the comparable program RNAalifold (which does not allow for user intervention) with different parameters to the selected 55 alignments (see Table S2). Mean prediction accuracies for the different alignments and program parameters are summarized in Fig. S5.

For ConStruct we tested several weighting factors for the thermodynamics and covariance (mutual information MI or RNAalifold covariation RCV) terms. Similarly, we varied the covariance term "cv" for RNAalifold, which adjusts the ratio between thermodynamics and covariance in RNAalifold's scoring function.

The average $MCC$ for the 55 alignments with ConStruct using only MI values ($w_{\mathrm{CV}} = 1.0$) was 20 % (see Fig. S5, column 1), but increased to 29 % by applying MI pair entropy normalization (Fig. S5, column 2). A combination of MI and thermodynamic base pairing probabilities led to a drastic improvement in prediction accuracy (Fig. S5, columns 3–6). Contrary to our expectations, however, the best result (mean $MCC = 0.82$, column 7) was obtained by ConStruct's predictions using only thermodynamic pairing probabilities. Only a slight improvement by about 0.5 % was obtained by a combination of thermodynamics and RCV (without or with stacking, respectively; columns 8 and 9).

In comparison, the average $MCC$ for the tool RNAalifold was lower by at least 10 %; the best result was obtained without use of RNAalifold's scoring function ($cv = 0$; column 12).

The low number of sequences—a mean value of only 14—in each sequence set might be responsible for the results with only MI in ConStruct and covariance in RNAalifold, respectively. Note, that in most real-world scenarios there are not more sequences available. Both tools compensate for this, however, by the use of thermodynamics.

In our evaluation we assume that the structures provided in the Rfam seed database are perfect. Rfam entries are annotated with either a status "predicted" (mostly by PFold) or "published". Our set of 55 entries consists of 13 with status "published" and 42 with status "predicted". The MCC for the "published" entries is 0.88 using only thermodynamics for prediction, whereas it is 0.80 for the "predicted" entries. That is, the "published" entries might be more trustworthy due to additional, experimental verification of the Rfam structures. A correlation between the pairwise sequence identity of the alignments and the quality of predicted consensus structures and/or number of sequences was not noticeable with the Rfam entries.

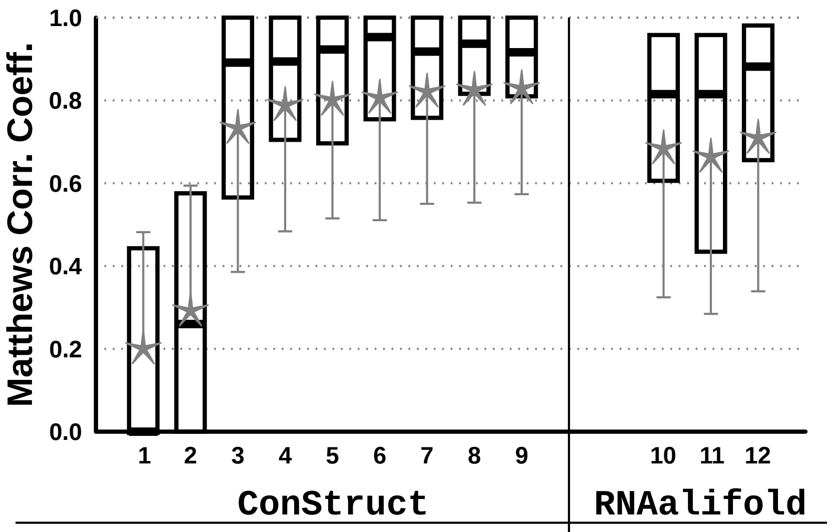


| | 1 | 2 | 3 | 4 | 5 | 6 | 7 | 8 | 9 | | 10 | 11 | 12 |
|---|---|---|---|---|---|---|---|---|---|---|---|---|---|
| $w_{thermo}$ | -- | -- | 0.75 | 0.7 | 0.7 | 0.7 | 1 | 0.7 | 0.7 | *cv* | 1.0 | 0.5 | 0.0 |
| $t_{thermo}$ | -- | -- | 0.1 | 0.03 | 0.03 | 0.03 | 0.03 | 0.03 | 0.03 | | | | |
| $w_{cv}$ | 1 | 1 | 0.25 | 0.3 | 0.3 | 0.3 | -- | 0.3 | 0.3 | | | | |
| $t_{cv}$ | 0.3 | 0.3 | 0.5 | 0.3 | 0 | 0.3 | -- | 0.3 | 0.3 | | | | |
| *pnorm* | -- | + | + | + | -- | -- | -- | -- | -- | | | | |

**Figure S5 - Prediction accuracy of ConStruct and RNAalifold for a set of 55 Rfam seed alignments.**

The consensus structures predicted by ConStruct and RNAalifold are compared to the corresponding Rfam structures as "reference" (see Table S2). The Matthews correlation coefficient (MCC) was computed by means of `compare_ct.pl` [4]. Lower, median and upper quartiles of MCC are shown as a box; the grey star and vertical line are mean and standard deviation, respectively. The table below the graph gives the parameters used for ConStruct's and RNAalifold's prediction. For an explanation of ConStruct's options and parameters see step 6 in section on "ConStruct's approach to consensus structure prediction" and equation for $P_c(i,j)$. The functions used to produce values of columns are:

1. ConStruct with only mutual information content
2. ConStruct with only (mutual information content plus normalization)
3. ConStruct with thermodynamics plus (mutual information content plus normalization)
4. ConStruct with thermodynamics plus (mutual information content plus normalization)
5. ConStruct with thermodynamics plus mutual information content
6. ConStruct with thermodynamics plus mutual information content
7. ConStruct with only thermodynamics
8. ConStruct with thermodynamics plus RNAalifold covariation
9. ConStruct with thermodynamics plus (RNAalifold covariation with stacking)
10. RNAalifold with default covariation term
11. RNAalifold with only 50 % covariation term
12. RNAalifold without covariation term

**Table S2 - The 55 alignments from Rfam database [1, 2] used for structure prediction.**

In the Rfam annotation, SS denotes how the consensus structure is derived: "pr" means predicted mostly by PFold [5], "pu" means published. The "structure conservation index" (SCI) is defined [6] as the ratio of the minimum free energy (MFE) of the consensus structure (as calculated by RNAalifold [7]) and the average MFE of the individual sequences in the alignment; a SCI value close to 1 indicates a perfectly conserved secondary structure; values above 1 are additionally supported by compensatory mutations. The "average pairwise sequence identity" (APSI)—a score for sequence conservation in an alignment—is computed by alistat, a program from the SQUID package [8]. The number of sequences in each alignment is given as "size". The Rfam alignments are available at http://www.biophys.uni-duesseldorf.de/construct3/paper2007suppl/rfam_ref_aln.tar.gz .

| # | Seq. name | ID (RF00#) | SS | SCI | APSI | Size |
|---|---|---|---|---|---|---|
| 1 | U28 | 085 | pr | 0.19 | 0.69 | 4 |
| 2 | U7 | 066 | pr | 0.0 | 0.7 | 30 |
| 3 | U18 | 093 | pr | 0.0 | 0.68 | 16 |
| 4 | IRES_EBNA | 448 | pu | 0.4 | 0.68 | 9 |
| 5 | U15 | 067 | pr | 0.1 | 0.6 | 18 |
| 6 | snoR31_Z110_Z27 | 353 | pr | 0.19 | 0.67 | 8 |
| 7 | snosnR60_Z15 | 309 | pr | 0.0 | 0.68 | 24 |
| 8 | U8 | 096 | pr | 0.15 | 0.67 | 6 |
| 9 | U43 | 221 | pr | 0.0 | 0.63 | 6 |
| 10 | RyhB | 057 | pr | 0.27 | 0.7 | 9 |
| 11 | U25 | 054 | pr | 0.0 | 0.62 | 8 |
| 12 | U14 | 016 | pr | 0.08 | 0.66 | 18 |
| 13 | U4 | 015 | pu | 0.53 | 0.66 | 26 |
| 14 | U3 | 012 | pu | 0.01 | 0.46 | 21 |
| 15 | ykoK | 380 | pr | 0.54 | 0.6 | 39 |
| 16 | U40 | 218 | pr | 0.26 | 0.69 | 9 |
| 17 | snoPyro_CD | 095 | pr | 0.0 | 0.59 | 38 |
| 18 | SraC_RyeA | 101 | pr | 0.28 | 0.7 | 7 |
| 19 | snoR38 | 213 | pr | 0.01 | 0.58 | 12 |
| 20 | ydaO-yuaA | 379 | pr | 0.34 | 0.56 | 35 |
| 21 | rne5 | 040 | pu | 0.78 | 0.65 | 7 |
| 22 | mir-192 | 130 | pr | 0.98 | 0.7 | 4 |
| 23 | FIE3 | 227 | pr | 0.37 | 0.65 | 5 |
| 24 | mir-1 | 103 | pr | 0.67 | 0.7 | 7 |
| 25 | msr | 170 | pr | 0.24 | 0.54 | 8 |
| 26 | Telomerase-cil | 025 | pu | 0.48 | 0.56 | 16 |
| 27 | mir-34 | 456 | pr | 0.8 | 0.66 | 8 |
| 28 | CsrB | 018 | pr | 0.52 | 0.65 | 9 |
| 29 | Purine | 167 | pu | 0.74 | 0.54 | 37 |
| 30 | 6S | 013 | pu | 0.75 | 0.65 | 7 |
| 31 | mir-7 | 053 | pr | 0.65 | 0.67 | 6 |
| 32 | mir-395 | 451 | pr | 0.45 | 0.66 | 28 |
| 33 | QaRNA | 388 | pu | 0.73 | 0.7 | 5 |
| 34 | mir-10 | 104 | pr | 0.29 | 0.58 | 11 |
| 35 | U5 | 020 | pu | 0.4 | 0.59 | 33 |
| 36 | glmS | 234 | pu | 0.72 | 0.55 | 14 |
| 37 | S15 | 114 | pu | 0.18 | 0.66 | 11 |
| 38 | mir-172 | 452 | pr | 0.58 | 0.62 | 8 |
| 39 | mir-8 | 241 | pr | 0.62 | 0.65 | 9 |
| 40 | mir-160 | 247 | pr | 0.63 | 0.66 | 7 |
| 41 | mir-46 | 249 | pr | 0.95 | 0.69 | 4 |
| 42 | U36 | 049 | pr | 0.0 | 0.63 | 20 |
| 43 | mir-2 | 047 | pr | 0.48 | 0.7 | 8 |
| 44 | mir-15 | 455 | pr | 0.98 | 0.69 | 4 |
| 45 | mir-399 | 445 | pr | 0.44 | 0.59 | 13 |
| 46 | let-7 | 027 | pr | 0.78 | 0.7 | 12 |
| 47 | lin-4 | 052 | pr | 0.65 | 0.69 | 9 |
| 48 | mir-166 | 075 | pr | 0.31 | 0.6 | 11 |
| 49 | Y | 019 | pu | 0.62 | 0.64 | 16 |
| 50 | RyeE | 112 | pr | 1.05 | 0.7 | 3 |
| 51 | U27 | 086 | pr | 0.13 | 0.69 | 10 |
| 52 | snoZ37 | 055 | pr | 0.0 | 0.64 | 8 |
| 53 | IRE | 037 | pu | 1.26 | 0.61 | 39 |
| 54 | ykkC-yxkD | 442 | pr | 0.84 | 0.61 | 16 |
| 55 | U54 | 206 | pr | 0.28 | 0.68 | 13 |
| | Mean #1–55: | | | 0.43 | 0.64 | 14 |

## 2 Influence of sequence numbers on prediction quality

The rational behind combining thermodynamics with covariation is to reduce the number of sequences necessary for for consensus structure prediction. Then, how many sequences are usually sufficient?

To get some insight into this problem, we selected a high-quality alignment of bacterial 16S rRNAs [9]. To reduce computing time and a possible influence of kinetics on folding, we restricted our analysis to domain I of 16S rRNA. The shortened alignment consisted of 371 non-identical sequences with APSI $\approx$ 0.75 and a mean length of 550 nt. From this "full" alignment we selected ten subalignments with 5, 10, and 20 different sequences, respectively.

For MCC calculations by means of `compare_ct.pl` [4] we compared the consensus structure predicted by ConStruct to a reference structure, the corresponding secondary structure of 16S rRNA from *E. coli*, which was extracted by rnaview [10] from the X-ray structure of an *E. coli* ribosome (PDB-ID 2AVY; [11]). The same procedure was applied to 100 different single sequences from the full alignment and to the full alignment. Results are shown in Table S3.

Using only thermodynamics for prediction, the MCC increased from 0.56 for single sequences to 0.83 for the full alignment (grey line in figure of Table S3), but the MCC was close to its maximal value already with 10 sequences. Using a combination of thermodynamics and MI ($w_{\mathrm{TD}} = 0.7$, $w_{\mathrm{CV}} = 0.3$; black line) led to a slight performance gain for the full alignment, i. e. with all available sequences. In contrast, using only the MI for predictions (dotted grey line) led to a much lower MCC even for the full alignment (MCC = 0.69).

Note, however, that for MI-only predictions the specificity (0.88 for alignments with 10 sequences and 0.91 for the full alignment) is above that of thermodynamics-only predictions (0.85 for alignments with $\geq$ 10 sequences), while the contrary is true for sensitivity (0.3 and 0.52 for MI-only and 0.81 for thermodynamics-only).

**Table S3 - Prediction accuracy of ConStruct in dependence upon number of sequences in an alignment.**

The given numbers for Matthews correlation coefficient (MCC), sensitivity (Sens.) and specificity (Spec.) are mean values for 100 (1 sequence) and 10 calculations (5–100 sequences), respectively.

The figure depicts MCC values up to alignments with 20 sequences. The stars give the MCC for the full alignment with 371 sequences, either predicted using only the MI (dotted grey), only thermodynamics (grey), or a combination of thermodynamics and MI (black).

| # seq.[a] | $w_{\mathrm{TD}} = 0.0$[b] | | | $w_{\mathrm{TD}} = 0.7$[c] | | | $w_{\mathrm{TD}} = 1.0$[d] | | |
|---|---|---|---|---|---|---|---|---|---|
| | MCC | Sens. | Spec. | MCC | Sens. | Spec. | MCC | Sens. | Spec. |
| 1 | 0. | 0. | 0. | 0.56 | 0.55 | 0.57 | 0.56 | 0.55 | 0.57 |
| 5 | 0.35 | 0.17 | 0.70 | 0.79 | 0.75 | 0.83 | 0.76 | 0.74 | 0.78 |
| 10 | 0.51 | 0.30 | 0.88 | 0.84 | 0.82 | 0.86 | 0.83 | 0.81 | 0.85 |
| 20 | 0.54 | 0.34 | 0.87 | 0.84 | 0.82 | 0.86 | 0.82 | 0.80 | 0.85 |
| 50 | 0.60 | 0.40 | 0.92 | 0.84 | 0.82 | 0.86 | 0.83 | 0.81 | 0.86 |
| 100 | 0.64 | 0.45 | 0.91 | 0.85 | 0.83 | 0.86 | 0.83 | 0.81 | 0.85 |
| 371 | 0.69 | 0.52 | 0.91 | 0.84 | 0.84 | 0.85 | 0.83 | 0.82 | 0.85 |

[a] Number of sequences in alignment
[b] Weighting with $w_{\mathrm{TD}} = 0.0$; $w_{\mathrm{CV}} = 1.0$, $t_{\mathrm{CV}} = 0.3$ (only mutual information content)
[c] Weighting with $w_{\mathrm{TD}} = 0.7$; $w_{\mathrm{TD}} = 0.03$, $w_{\mathrm{CV}} = 0.3$, $t_{\mathrm{CV}} = 0.3$
(70 % thermodynamics plus 30 % mutual information content)
[d] Weighting with $w_{\mathrm{TD}} = 1.0$, $t_{\mathrm{TD}} = 0.03$; $w_{\mathrm{CV}} = 0.0$ (only thermodynamics)

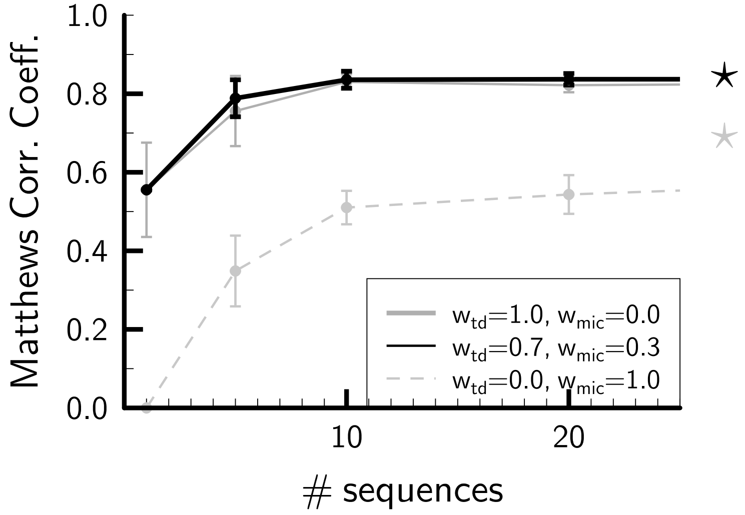

## 3 Refinement of an initial alignment using ConStruct

In Fig. S6 the process of manually refining a sequence alignment is shown. The initial alignment of 14 SECIS sequences was created by means of MAFFT [12] (without special options); the resulting sequence alignment is shown in Fig. S7. This alignment is used by CS_FOLD to calculate structure distribution files *via* RNAFOLD and to write the project file. After loading the project file with CS_DP, the consensus dotplot (Fig. S6A left) is shown. According to the dotplot most sequences are structurally aligned; that is, single sequences base pairs (green and blue squares) are superimposed and build the red consensus base pairs. Moving the mouse cursor over the green square at basepair position 33:5 in the dotplot (see right arrow) highlights nucleotides 5 and 33 of sequence `M_kandleri_hdr_A`; moving the mouse cursor over the green square at basepair position 23:18 in the dotplot (see left arrow) highlights nucleotides 18 and 23 of the same sequence. Clicking to nucleotide 33 of `M_kandleri_hdr_A` in the alignment editor selects this sequence; note the blue squares in the dotplot of Fig. S6B. Clicking to nucleotide 23 of `M_kandleri_hdr_A` selects the subsequence 23–33; note the blue background of the subsequence in the alignment editor. Clicking five times with the right mouse button to the double-headed arrow (top-line of alignment editor) moves the selected subsequence five positions towards the 3' end; thereby the five gaps, which are located 3' of the subsequence prior to its movement, are shifted to the 5' end of the subsequence. After selection of the subsequence 1–22 of `M_kandleri_hdr_A` and moving it by one position rightwards (Fig. S6C), three basepairs of the distal helix (close to the hairpin loop) contribute to the consensus pairs; the proximal helix is located close to the remaining consensus pairs.

Now the two gaps at positions 4–5 are obviously unnecessary: clicking position 1 of `M_jannaschii_sps` (first sequence) and position 3 of `M_kandleri_fmfdh_B` (third sequence) and moving the three subsequences by three position rightwards results in the dotplot and alignment shown in Fig. S6D; clicking position 1 of `M_kandleri_sps` (fifth sequence) and to position 3 of `M_jannaschii_fmfdh_B` (last sequence) and moving the ten subsequences by three position rightwards results in the dotplot and alignment shown in Fig. S6E.

Except the sequence range 19–28, which forms the hairpin loop, the structural alignments shown in Fig. S6(bottom) is close to the alignment shown in Fig. 2B.

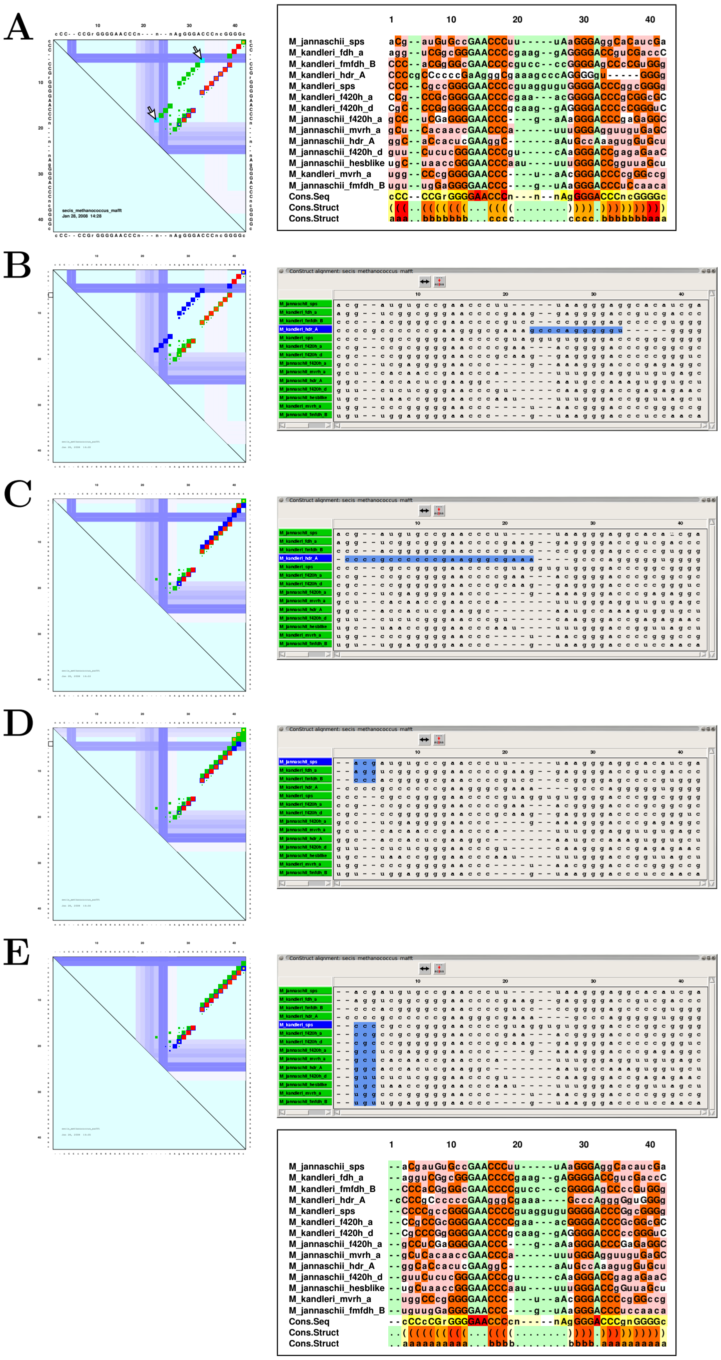


**Figure S6 - Refinement of an initial alignment using ConStruct.**
For details see text on previous page.

```
> M_jannaschii_sps
acg--augugccgaacccuu-----uaagggaggcacaucga
> M_kandleri_fdh_a
agg--ucggcgggaaccccgaag--gaggggaccgucgaccc
> M_kandleri_fmfdh_B
ccc--acggggcgaaccccgucc--ccggggagccccguggg
> M_kandleri_hdr_A
ccccgccccccgaagggcgaaagcccagggggu-----gggg
> M_kandleri_sps
ccc--cgccggggaaccccguaggugugggggacccggcgggg
> M_kandleri_f420h_a
ccg--ccgcggggaaccccgaa---acggggacccgcggcgc
> M_kandleri_f420h_d
cgc--ccgggggggaacccgcaag--gaggggacccccggguc
> M_jannaschii_f420h_a
gcc--ucgaggggaaccc----g--aaagggacccgagaggc
> M_jannaschii_mvrh_a
gcu--cacaaccgaaccca------uuugggagguugugagc
> M_jannaschii_hdr_A
ggc--accacucgaaggc-------aaugccaaaguggugcu
> M_jannaschii_f420h_d
guu--cucucgggaacccgu-----caagggaccgagagaac
> M_jannaschii_hesblike
ugc--uaaccgggaacccaau----uuugggaccgguuagcu
> M_kandleri_mvrh_a
ugg--cccgggggaaccc----u--aacgggaccccgggccg
> M_jannaschii_fmfdh_B
ugu--uggaggggaaccc----g--uaagggacccuccaaca
```

**Figure S7 - Sequence alignment of SECIS sequences created by mafft.** With this alignment the project depicted in Fig. S6A was created.

### Table S4 - Colors used by ConStruct.

| Color | Description |
|---|---|
| | **GUI: Upper-right triangle of dotplot** |
| | Background |
| | Base pair of unselected sequence[1] |
| | Base pair of selected sequence[1] |
| | Consensus base pair[1] |
| | Percentage of gaps in alignment column[2] |
| | Partner[3] |
| | **GUI: Lower-left triangle of dotplot** |
| | MIC or RNAalifold score[4] |
| | **GUI: Alignment editor** |
| **Sequence name** | Name field of unselected sequence |
| **Sequence name** | Name field of selected sequence |
| | Range of nucleotides selected for move |
| | Base pair probability in individual sequences[5] |
| | **DrawStructure and Circles output** |
| | Consensus base pair probability |
| | **Structural alignment output** |
| | Mismatch contradicting the consensus base pair |
| | Covariation supporting the consensus base pair |
| | Base pair in accordance to consensus base pair |
| | Single-stranded region |
| | Consensus nucleotide probability (majority rule)[6] |
| | Consensus base pair probability[7] |

[1] Area of square is proportional to (sum of) thermodynamic pairing probability
[2] From 1 gap up to 100 % gaps in respective alignment column
[3] If the mouse points to a nucleotide, which is involved in at least one base pair, all corresponding base pairs are highlighted in the dotplot
[4] Selectable scaling of rainbow colors from $t_{\mathrm{CV}}$ to $\max(\mathrm{CV}_{ij})$
[5] Nucleotides in an alignment column are colorized if mouse points to a corresponding consensus base pair in the dotplot
[6] Line labeled "Cons.Seq"
[7] Line labeled "Cons.Struct"

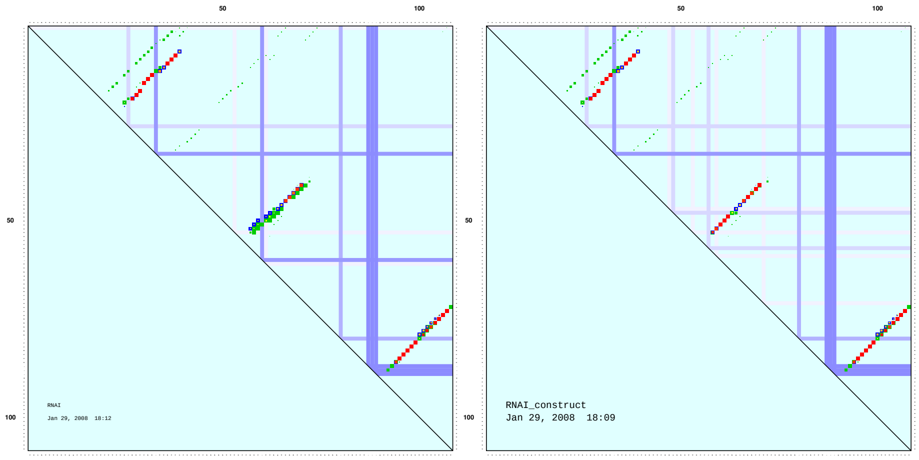


```
> Y17716_1_6521-6420
CGUAUUUGGUGGCAGCGGUCUUGUACACCCUG-UUACCACGGUUCAGAAGUUCCCCAAC-
UUACUGAACCUUCGAAAAA-CCACCU---CCCCAGGUGGUUUUUUCGU
> AJ132618_1_824-723
CGAAUUUGGUGGCAGCGGUCUUGUACACCCUG-UUACCACGGUUCAGAAGUUCCCCAAC-
UUACUGAACCUUCGAAAAA-CCACCU---CCCCAGGUGGUUUUUUCGU
> X63534_1_2865-2765
UAGAUUUGGUUACUGCGCUCCUCCA-AGCCAG-UUACCGCGGUUCAAAGAGUUGGUAGC-
UCAGAGAACCUUCGAAAAA-CCGCCU---UGCAGGGCGGUUUUUUCGU
> U65460_1_39-143
AGUAUUUGGUAUCUGUGCUCUGCUGAAGCCAGAUUACCUCGGUUAGAUAAUCUGCCAGGA
UUAGCUAACCUUCGAAAAAACCACCU---GCCAGGGUGGUUUUUUCGU
> D21263_1_151-50
CGAAUUUGGUGGCAGCGGUCUUGUACACCCUG-UUACCACGGUUCAUAAACUUGCCGGU-
UUAAUGAACCUUCGAAAAA-CCACCU---UGCCGGGUGGUUUUUUCGU
> X80302_1_245-141
AGUAUUUGGUAUCUGUGCUCUGCUGAAGCCAGAUUACCUCGGUUAGAUAAUCUGCCAGGA
UUAGCUAACCUUCGAAAAAACCACCU---GCCAGGGUGGUUUUUUCGU
> U80803_1_804-704
CAGAUUUGGUUACUGCGUUCCUCCA-AGCCAG-UUACCGCGGUUCAAAGAGUUGGUAGC-
UCUGAGAACCUUCGAAAAA-CCGCCU---UGCAGGGCGGUUUUUUCGU
> AF156893_2_4598-4498
CGAAUUUGGUUACUGCGCUCCUCCA-AGCCAG-UUACCUCGGUUCAAAGAGC-GCCAAC-
UCCUCGAACCUUCGAAAAAACCGCCC---GCCAAGGCGGUUUUUUCGU
> S42973_1_3-107
AGUAUUUGGUAUCUGCGCUCUGCUGAAGCCAG-UUACCUUCGGAAAAAGAGUUGGUAGC-
-UCUUGAUCCGGCAAACAAACCACCGCUGGUAGCGGUGGUUUUUUUGU
> Y17846_1_1185-1085
-AGAUUUGGUGGCAGCGGUCUUGUACACCCUG-UUACCACGGUUCAGAAGUUCCCCAAC-
UUACUGAUCCUUCGAAAAA-CCACCU---CCCCAGGUGGUUUUUUCGU
```

```
> Y17716_1_6521-6420
CGUAUUUGGUGGCAGCGGUCUUGUACACCCUG-UUACCACGGUUCAG-AAGUUCCCCAAC
UUACUGAACCUUCGAAAAA-CCACCU---CCCCAGGUGGUUUUUUCGU
> AJ132618_1_824-723
CGAAUUUGGUGGCAGCGGUCUUGUACACCCUG-UUACCACGGUUCAG-AAGUUCCCCAAC
UUACUGAACCUUCGAAAAA-CCACCU---CCCCAGGUGGUUUUUUCGU
> X63534_1_2865-2765
UAGAUUUGGUUACUGCGCUCCUCCA-AGCCAG-UUACCGCGGUUCAAAGAGUUGGU-AGC
UCAGAGAACCUUCGAAAAA-CCGCCU---UGCAGGGCGGUUUUUUCGU
> U65460_1_39-143
AGUAUUUGGUAUCUGUGCUCUGCUGAAGCCAGAUUACCUCGGUUAGAUAAUCUGCCAGGA
UUAGCUAACCUUCGAAAAAACCACCU---GCCAGGGUGGUUUUUUCGU
> D21263_1_151-50
CGAAUUUGGUGGCAGCGGUCUUGUACACCCUG-UUACCACGGUUCA-UAAACUUGCCGGU
UUAAUGAACCUUCGAAAAA-CCACCU---UGCCGGGUGGUUUUUUCGU
> X80302_1_245-141
AGUAUUUGGUAUCUGUGCUCUGCUGAAGCCAGAUUACCUCGGUUAGAUAAUCUGCCAGGA
UUAGCUAACCUUCGAAAAAACCACCU---GCCAGGGUGGUUUUUUCGU
> U80803_1_804-704
CAGAUUUGGUUACUGCGUUCCUCCA-AGCCAG-UUACCGCGGUUCAAAGAGUUGGU-AGC
UCUGAGAACCUUCGAAAAA-CCGCCU---UGCAGGGCGGUUUUUUCGU
> AF156893_2_4598-4498
CGAAUUUGGUUACUGCGCUCCUCCA-AGCCAG-UUACCUCGGUUCAAAGAGC-GCCAA-C
UCCUCGAACCUUCGAAAAAACCGCCC---GCCAAGGCGGUUUUUUCGU
> S42973_1_3-107
AGUAUUUGGUAUCUGCGCUCUGCUGAAGCCAG-UUACCUUCGGAAAAAGAGUUGGU-AGC
UCUUGAUCCG-GCAAACAAACCACCGCUGGUAGCGGUGGUUUUUUUGU
> Y17846_1_1185-1085
-AGAUUUGGUGGCAGCGGUCUUGUACACCCUG-UUACCACGGUUCAG-AAGUUCCCCAAC
UUACUGAUCCUUCGAAAAA-CCACCU---CCCCAGGUGGUUUUUUCGU
```

**Figure S8 - Dotplots of RNAI sequences from Rfam (RF00106).**
The left dotplot is for the original seed alignment from Rfam 8.1; the right dotplot is for an alignment optimized with ConStruct. Note the non-superimposed base pairs in the Rfam alignment, which form a consensus helix (red) after correction. Below are the corresponding sequence alignments given.

## References


1. Griffiths-Jones S, Bateman A, Marshall M, Khanna A, Eddy S: **Rfam: an RNA family database.** *Nucleic Acids Res.* 2003, **31**:439–441.

2. Griffiths-Jones S, Moxon S, Marshall M, Khanna A, Eddy S, Bateman A: **Rfam: annotating non-coding RNAs in complete genomes.** *Nucleic Acids Res.* 2005, **33**:D121–D124.

3. Doshi K, Cannone J, Cobaugh C, Gutell R: **Evaluation of the suitability of free-energy minimization using nearest-neighbor energy parameters for RNA secondary structure prediction**. *BMC Bioinformatics* 2004, **5**:105.

4. Gardner P, Giegerich R: **A comprehensive comparison of comparative RNA structure prediction approaches.** *BMC Bioinformatics* 2004, **5**:140.

5. Knudsen B, Hein J: **Pfold: RNA secondary structure prediction using stochastic context-free grammars.** *Nucleic Acids Res.* 2003, **31**:3423–3428.

6. Washietl S, Hofacker IL, Stadler PF: **Fast and reliable prediction of noncoding RNAs.** *Proc. Nat. Acad. Sci. U.S.A.* 2005, **102**:2454–2459.

7. Hofacker IL, Fekete M, Stadler PF: **Secondary structure prediction for aligned RNA sequences.** *J. Mol. Biol.* 2002, **319**:1059–1066.

8. Eddy SR: **SQUID - C function library for sequence analysis.** 2005. [http://selab.wustl.edu/cgi-bin/selab.pl?mode=software#squid].

9. Lescoute A, Leontis N, Massire C, Westhof E: **Recurrent structural RNA motifs, Isostericity Matrices and sequence alignments.** *Nucleic Acids Res.* 2005, **33**:2395–2409.

10. Yang H, Jossinet F, Leontis N, Chen L, Westbrook J, Berman H, Westhof E: **Tools for the automatic identification and classification of RNA base pairs.** *Nucleic Acids Res.* 2003, **31**:3450–3460.

11. Schuwirth B, Borovinskaya M, Hau C, Zhang W, Vila-Sanjurjo A, Holton J, Cate J: **Structures of the bacterial ribosome at 3.5 Å resolution.** *Science* 2005, **310**:793–795.

12. Katoh K, Toh H: **Four-way consistency: an objective function of multiple ncRNA sequence alignment that considers predicted secondary structures.** *Nucleic Acids Res.* 2007, **in press**.
